# Changes in myocardial blood flow and microvascular resistance in patients with coronary artery disease undergoing transcatheter aortic valve implantation

**DOI:** 10.1136/openhrt-2025-003621

**Published:** 2025-12-30

**Authors:** Eron Yones, Rebecca Gosling, Daniel Taylor, Tom Alexander Howard Newman, Mark Sammut, Saadia Aslam, Javaid Iqbal, Muhammad Aetesam-ur-Rahman, Kenneth Morgan, Amir Aziz, Melanie Neville, Ever Grech, Paul D Morris, Julian Gunn

**Affiliations:** 1 School of Clinical Medicine and Population Health, The University of Sheffield, Sheffield, England, UK; 2 Sheffield Teaching Hospitals NHS Foundation Trust, Sheffield, UK; 3 The University of Sheffield, Sheffield, UK

**Keywords:** CORONARY ARTERY DISEASE, CORONARY PHYSIOLOGY, Aortic Valve Stenosis, Heart Valve Prosthesis

## Abstract

**Background:**

Severe aortic stenosis (AS) causes a pathophysiological cascade, which impairs myocardial blood flow. This effect is exacerbated in the presence of coronary disease (CAD). Treatment with transcatheter aortic valve implantation (TAVI) may promote reversal of these pathophysiological conditions.

**Methods:**

We performed multimodality assessment of cardiac physiology in patients with AS and concurrent CAD requiring percutaneous coronary intervention, prior to and 6 months after undergoing TAVI. Techniques include: coronary angiography and bolus thermodilution-derived indices of microvascular function (coronary flow reserve (CFR); index of microcirculatory resistance (IMR)); stress perfusion cardiac magnetic resonance (CMR) imaging, which was used to measure changes in global myocardial blood flow (MBF) and left ventricular mass (LVM), and computed resting and hyperaemic vessel specific absolute coronary flow (aCBF) and microvascular resistance (MVR) using a computational model of coronary physiology.

**Results:**

Data were obtained for seven patients (10 vessels). CFR increased from 1.53 (1.2–1.7) to 2.35 (2.0–2.7) (p=0.037) 6 months post-TAVI. There was a 33% reduction in resting aCBF from 218 mL/min to 146 mL/min (p=0.004). On CMR, resting MBF fell 37% from 3.0±0.98 mL/min/g to 1.9±0.7 mL/min/g (p=0.033) and stress MBF fell 25% from 3.6±0.57 mL/min/g to 2.7±0.7 mL/min/g (p=0.004). Indexed LVM regressed from 79±14 g/m^2^ to 71±16 g/m^2^ (p=0.006). MVR remained unchanged.

**Conclusions:**

CFR increased following TAVI. The mechanism for this was a significant reduction in resting coronary blood flow measured with CMR and modelled computationally. The unchanged MVR and IMR suggest that resting blood flow reduces due to reduced myocardial demand and myocardial remodelling, rather than changes in resistance.

**WHAT IS ALREADY KNOWN ON THIS TOPIC**

- Patients with severe aortic stenosis (AS) undergoing transcatheter aortic valve implantation (TAVI) frequently have concomitant coronary artery disease (CAD). The effect of TAVI on coronary physiology, including microvascular resistance and absolute myocardial blood flow, is relatively unknown.

**WHAT THIS STUDY ADDS**

- This study quantified changes in global and vessel-specific coronary physiology prior to and 6 months after TAVI in patients with AS and concomitant CAD requiring percutaneous coronary intervention. There was a significant increase in coronary flow reserve, primarily attributed to a reduction in resting coronary blood flow and myocardial remodelling.

**HOW THIS STUDY MIGHT AFFECT RESEARCH, PRACTICE OR POLICY**

- This study used advanced computational modelling to suggest a mechanism for the physiological response to TAVI in patients with AS. The role and treatment thresholds for coronary physiological indices in patients requiring TAVI are poorly understood, and these pilot data may inform the design of larger observational studies.

## Introduction

 Untreated severe aortic stenosis (AS) causes an unrelenting pathophysiological cascade which, over time, impairs myocardial blood flow (MBF) and coronary flow reserve (CFR).^1^ In health, tightly controlled autoregulatory mechanisms augment MBF during rest and stress.^2^ Patients with severe AS develop left ventricular hypertrophy (LVH) as a compensatory mechanism to increase LV contractile force to maintain adequate stroke volume against progressively increasing LV afterload and end-diastolic pressures (LVEDP).^1^ These haemodynamic conditions are compounded by high extravascular compressive forces and falling proximal perfusion pressures, resulting in systolic flow reversal of the epicardial:endocardial MBF ratio, which, in health, is approximately 1.2:1.^3^ This can result in subendocardial ischaemia and myocardial fibrosis even in the absence of coronary artery disease (CAD).^3^ Progressive LVH produces an LV with a relative paucity of capillaries and reduced diastolic filling time, reducing myocardial oxygen supply and creating a supply:demand mismatch at rest.^4^ In response, autoregulatory mechanisms that augment MBF during stress are upregulated through vasodilation of myocardial arterioles and capillaries, resulting in high resting MBF.^3^ Progressive exhaustion of myocardial vasodilatory capacity results in a supply:demand mismatch at times of stress with impaired CFR and vulnerability to ischaemia when myocardial oxygen demand rises.^3^ These mechanisms explain angina and myocardial ischaemia in severe AS despite absence of CAD.^1 5^

A number of small observational studies have prospectively measured indices of coronary physiology before and after transcatheter aortic valve implantation (TAVI) in patients without significant CAD to determine whether the complex and hostile haemodynamic conditions of severe AS regress with treatment of the diseased valve.^6^,^9^ The proportion of patients with concurrent CAD undergoing TAVI rises with age and risk category but has consistently been shown to be at least 50%.^10 11^ The issue of revascularisation with percutaneous coronary intervention (PCI) in these patients is not comprehensively resolved.^12^,^14^ We therefore aimed to produce a comprehensive multimodality assessment of MBF and microvascular disease (MVD) in patients with severe AS and concurrent CAD undergoing TAVI. We measured a series of conventional indices of coronary physiology and MVD, as well as absolute coronary and myocardial blood flow (aCBF) (using computational fluid dynamics (CFD) technology)^15^ and quantitative stress perfusion cardiac-MRI (CMR). We measured these parameters before and 6 months after treatment with TAVI. We hypothesised that treatment with TAVI would produce beneficial haemodynamic effects on the myocardium and microvasculature with positive LV remodelling in patients with revascularised CAD.

## Methods

### Design, ethics and patient population

This was a single centre, prospective, longitudinal, observational study undertaken at Sheffield Teaching Hospitals (STH) and The University of Sheffield. Patients listed for elective TAVI with >50% coronary artery stenosis in at least one major vessel were considered suitable for inclusion and were identified at the TAVI Heart Team meeting. They had standard indications for TAVI, invasive coronary angiography and PCI in accordance with European Society of Cardiology (ESC) and European Association for Cardiothoracic Surgery guidance.^16 17^ Exclusion criteria included <50% visual coronary artery stenosis and co-existing terminal illness. Invasive angiography and coronary physiology were undertaken as standard of care ‘work-up’ investigations prior to TAVI. Patients had the same set of physiology taken at baseline, after PCI (where appropriate) and again 6 months after TAVI. CMR was performed at least 2 weeks post-PCI (pre-TAVI) and 6 months post-TAVI.

All patients provided written informed consent. The study protocol and ethical approval were granted by the NHS research ethics committee and health research authority (22/NW/0017) and were compliant with the Declaration of Helsinki.

### Patient and public involvement

The entire VIRTUheart Programme, which started in 2009, is reviewed annually by the Sheffield National Institute of Health Research Cardiovascular Patient Panel. They reviewed the study protocol, advised how to best approach potential participants and gave suggestions for the patient information sheet and the consent form.

### Coronary angiography and physiology

All procedures adhered to standard clinical protocols and ESC guidance for investigation and management of CAD.^17^ Vascular access was achieved through the radial artery in preference to the femoral artery with a 6Fr peripheral sheath. Images were obtained using 6Fr guiding catheters. Patients received 70–100 IU/kg of unfractionated heparin, administered intra-arterially to achieve an activated clotting time of 250–350 s. Image acquisition was performed using a predefined protocol for CFD modelling, which required good vessel opacification, minimal overlap, magnification, panning and at least three views of the right and five of the left coronary arteries.^15^ Any visually intermediate coronary stenoses (50%–90% stenosis) were subject to physiology assessment according to clinical protocols.^18^ Patients underwent a full invasive pressure wire study with a 0.014” pressure-temperature sensor-tipped Abbott Pressure-Wire^TM^ X (Abbott Vascular, Santa Clara, CA, USA) which was zeroed outside of the body and equalised to central aortic pressure in the aortic root. The wire was advanced carefully beyond the coronary stenosis to the distal coronary artery. Trans-lesional data were transmitted from the transducer near the distal end of the wire to Coroflow^TM^ software (Coroventis, Uppsala, Sweden) using wireless technology. Trans-lesional pressure and temperature data were obtained at baseline and during hyperaemia, the latter of which was induced with an intravenous Adenosine infusion given at a rate of 140 ug/kg/min. Trans-lesional pressure ratio (P_d_/P_a_) was assessed under baseline conditions to measure resting full-cycle ratio (RFR) and hyperaemia to measure fractional flow reserve (FFR). Bolus thermodilution, with 3 mL of room temperature 0.9% saline, was performed at baseline and during hyperaemia to measure CFR and index of microcirculatory resistance (IMR). CFR was calculated as the ratio between the mean transit times of three boluses of saline at rest and during hyperaemia. IMR was calculated as the mean P_d_ multiplied by the thermodilution-derived mean transit time of a bolus of saline during hyperaemia. Microvascular resistance reserve (MRR) was calculated using the formula *MRR = (CFR/FFR) x (Pa. rest / Pa. hyperaemia*).^19^ If the FFR was ≤0.80, PCI was performed according to current ESC guidance.^17^ This was performed at the same sitting and within local guidance and protocols. Latest generation drug-eluting stents were used. In any treated vessel, the full range of coronary physiology was repeated to ensure adequate revascularisation had been achieved and accurate up-to-date data were obtained for comparison post-TAVI. This protocol was used for both the pre- and post-TAVI angiogram and pressure wire assessments, and the same set of data was obtained each time.

### Computational coronary physiology

This study used the virtuQ^TM^ (v4.0.0.6) CFD software package, developed at the University of Sheffield, to perform offline computation of aCBF and microvascular resistance (MVR).^15 20^ The virtuQ^TM^ workflow uses angiographic images to reconstruct a patient-specific in silico 3D coronary artery and applies invasively measured hyperaemic and resting P_d_/P_a_ values to derive aCBF (in mL/min) and MVR (in mm Hg.min/mL). Both indices were calculated under hyperaemic and resting conditions. The virtuQ^TM^ methodology and validation studies have been reported previously.^15 20^ In brief, reconstructions used two angiographic projections, acquired at least 30° apart and during end-diastole, which were semi-automatically segmented. The reconstruction inlet and outlet corresponded to the locations of proximal and distal invasive pressure assessment, respectively. Side branch flow to unresolved vessels was inferred from local taper of the reconstruction,^21^ with a flow-diameter scaling exponent of 2.33.^22 23^ All simulations used standard parameters for blood viscosity, µ=0.0035 Pa s and density ρ=1050 kg/m^3^. Online supplemental figure 1 demonstrates an example of the virtuQ^TM^ workflow.

### Cardiac MRI

Patients underwent stress perfusion CMR on a 1.5 Tesla GE 450W whole body MRI scanner (GE Healthcare, Milwaukee, Wisconsin). Patients were instructed to refrain from caffeine for 24 hours before the study. Standard safety procedures were followed, and all patients completed a pre-scan safety questionnaire. The CMR protocol included a baseline survey, cine imaging (vertical long axis (two chambers), horizontal long axis (four chambers) and short axis contiguous left ventricular volume stack, acquired using balanced steady-state free precession in a single slice breath hold sequence), stress and rest perfusion imaging and 2D late gadolinium enhancement imaging. Intravenous regadenoson (400 ug) was used as the stressor agent.

Image analysis and reporting was performed offline using MASS research reporting software (Version 2023 EXP, Leiden University Medical Centre, NL). To compute LV volumes, LV mass (LVM) and LV function, the epicardial and endocardial borders on the short axis stack were manually traced excluding papillary muscles. Values were indexed to body surface area (BSA) where appropriate. Quantitative perfusion analysis was performed using a single bolus, single sequence technique. Endocardial and epicardial contours were drawn manually, automatically propagated and manually corrected when necessary. Right ventricular insertion points were marked, and a 16 segment American Heart Association model was used. Absolute myocardial blood flow (ml/min/g) was quantified using a Fermi function constrained deconvolution method as described previously.^24 25^ Mean MBF was produced in mL/min/g and was multiplied by LVM to produce global MBF in mL/min. Myocardial perfusion reserve (MPR) was calculated as the ratio of global MBF at stress to resting global MBF.

### Statistical analysis

Continuous data are presented as mean values with SD or median values with IQR. Categorical data are presented as counts (n) and percentage values. The Shapiro-Wilk test was used to determine normality of data. Continuous paired data points were analysed with paired t-tests or Wilcoxon signed rank tests as appropriate. A two-tailed P value of <0.05 was considered significant. Statistical analysis and charts were produced using SPSS (V.29, IBM, NY, USA).

## Results

A total of 22 patients were recruited into the study and seven patients returned for follow-up angiography and physiology 6 months post-TAVI (see supplementary material for consort diagram). The mean age was 83±6 years, and the majority were male. Table 1 summarises baseline patient characteristics and comorbidities. From these seven patients, data were obtained from 10 coronary arteries. Of these, seven were the left anterior descending artery (LAD), with two right coronary arteries (RCA) and one circumflex (LCx). All patients (eight vessels) received PCI to at least one vessel at the index procedure (six LAD and two RCA). No patients had PCI post-TAVI. The same patients had pre- and post-TAVI CMR.

**Table 1:** Clinical characteristics

Clinical characteristics (n=7)	Baseline	Post-TAVI
Age	83±6	–
Male sex *n (%*)	6 (86%)	–
Female sex *n (%*)	1 (14%)	–
Weight (kg)	85±20	–
BMI	30±5.3	–
Heart rate (bpm)	69±9	67±8
Systolic BP	141±12	138±11
Diastolic BP	85±6	82±6
Comorbidities		
Hypertension *n (%*)	3 (43%)	–
Hyperlipidaemia *n (%*)	6 (86%)	–
History of smoking *n (%*)	1 (14%)	–
Atrial fibrillation *n (%*)	1 (14%)	–
Pacemaker *n (%*)	1 (14%)	–
Peripheral vascular disease *n (%*)	1 (14%)	–
Previous stroke or TIA *n (%*)	3 (43%)	–
Medication		
Aspirin *n (%*)	7 (100%)	7 (100%)
P2Y12 inhibitor *n (%*)	5 (71%)	5 (71%)
DOAC *n (%*)	2 (28%)	2 (28%)
Statin *n (%*)	5 (71%)	5 (71%)
Beta-blocker *n (%*)	3 (43%)	4 (57%)
ACE-inhibitor *n (%*)	3 (43%)	3 (43%)
Calcium channel blocker *n (%*)	1 (14%)	1 (14%)
Loop diuretic *n (%*)	5 (71%)	5 (71%)
Clinical scores		
Rockwood frailty score	4 (IQR 4–6)	–
New York Heart Association Score	3.0±0.5	–

Data are presented in counts and percentages, mean±SD or median with IQR.

ACE, angiotensin converting enzyme; BMI, body mass index; BP, blood pressure; DOAC, direct oral anti-coagulant; TAVI, transcatheter aortic valve implantation; TIA, Transient Ischaemic Attack.

### Changes in invasive and computed coronary physiology

The CFR did not change following PCI but there was a significant improvement post-TAVI (1.53 (1.2–1.7) to 2.35 (2.0–2.7), p=0.037). The FFR increased significantly both post-PCI and post-TAVI, and all but one patient had an FFR >0.80 pre-TAVI (table 2). The single FFR of 0.77 pre-TAVI increased to 0.85 post-TAVI. RFR and IMR did not change at follow-up. There was a significant increase in the MRR (table 2). Baseline vessel inlet aCBF increased significantly post-PCI but fell back to a similar level post-TAVI (149±35 mL/min vs 218±90 mL/min vs 146±89 mL/min, p=0.004) (table 2). Hyperaemic aCBF improved significantly after PCI, which was maintained post-TAVI. Computed MVR remained similar at post-PCI and at follow-up.

**Table 2:** Changes in invasive and computed coronary physiology at baseline, post-PCI and post-TAVI

	Baseline n=10	Pre-TAVI (post-PCI) n=10	P value	Post-TAVI n=10	P value
P_d_ rest (mm Hg)	67±21	84±18	*0.034*	83±16	*0.18*
P_a_ rest (mm Hg)	89±18	91±18	*0.74*	88±17	*0.39*
P_a_/P_d_ rest (mm Hg)	0.81 (0.7–0.86)	0.91 (0.89–0.96)	*0.015*	0.95 (0.91–0.98)	*0.052*
RFR	0.68 (0.52–0.75)	0.89 (0.84–0.94)	*0.003*	0.92 (0.90–0.96)	*0.13*
P_d_ hyperaemia (mm Hg)	49±19	67±16	*0.007*	68±16	*0.69*
P_a_ hyperaemia (mm Hg)	72±21	77±15	*0.21*	77±18	*0.98*
FFR	0.74 (0.66–0.75)	0.86 (0.82–0.90)	*0.005*	0.89 (0.86–0.92)	*0.032*
CFR	1.2 (1.1–1.5)	1.53 (1.2–1.7)	*0.61*	2.35 (2.0–2.7)	*0.037*
IMR (mm Hg.sec)	22±14	24±12	*0.72*	26±15	*0.56*
MRR	2.3 (1.9–2.7)	2.0 (1.3–2.8)	*0.17*	3.2 (2.3–3.4)	*0.009*
Baseline vessel inlet flow (mL/min)	149±35	218±90	*<0.001*	146±89	*0.004*
Baseline vessel outlet flow (mL/min)	58±29	66±42	*0.337*	50±32	*0.07*
Baseline MVR (mm Hg.min/mL)	1.2 (0.5–1.5)	1.3 (0.9–2.2)	*0.855*	2.3 (1.2–3.6)	*0.092*
Hyperaemic vessel inlet flow (mL/min)	170±28	273±104	*0.004*	215±66	*0.13*
Hyperaemic vessel outlet flow (mL/min)	66±30	94±58	*0.224*	87±42	*0.71*
Hyperaemic MVR (mm Hg.min/mL)	0.56 (0.4–1.3)	0.91 (0.5–1.3)	*0.438*	0.9 (0.8–1.0)	*0.86*

Data are presented as mean±SD or median and IQR. Baseline flow in: coronary inlet flow. Baseline flow out: coronary flow distal to stenosis; .

The first p value column demonstrates the p value between mean pre- and post-PCI values, and the second p value column represents p values between post-PCI and 6 months post-TAVI mean values.

CFR, coronary flow reserve; FFR, fractional flow reserve; IMR, index of microcirculatory resistance; MRR, microvascular resistance reserve; MVR, microvascular resistance; P_a_, aortic pressure; PCI, percutaneous coronary intervention; P_d_, distal coronary pressure; RFR, resting full-cycle ratio; TAVI, transcatheter aortic valve implantation.

### Changes in cardiac-MRI-derived physiology

There was a significant reduction in LVM indexed to BSA (LVMi) and hyperaemic and resting MBF post-TAVI (table 3). No other parameters measured on CMR changed significantly at follow-up.

**Table 3:** Changes in cardiac MRI derived parameters of myocardial function and blood flow pre- and post-TAVI

	Pre-TAVI n=7	Post-TAVI n=7	P value
LVEF (%)	58±12	57±10	*0.98*
LV EDVi (mL/m^2^)	92±14	83±14	*0.17*
LV ESVi (mL/m^2^)	38±13	36±13	*0.18*
SVi (mL/m^2^)	52±13	47±6	*0.32*
CI (L/min/m^2^)	3.2±0.6	2.9±0.5	*0.28*
LVM ED (g)	163±26	142±31	*0.004*
LVMi (g/m^2^)	82±14	71±16	*0.006*
LVM/LVEDV	0.90±0.17	0.87±0.15	*0.57*
MBF at rest/g (mL/min/g)	3.0±0.98	1.9±0.7	*0.033*
Total MBF at rest (mL/min)	485±141	265±95	*<0.001*
MBF at stress/g (mL/min/g)	3.6±0.57	2.7±0.7	*0.004*
Total MBF at stress (mL/min)	590±119	378±120	*<0.001*
MPR	1.1 (1.0–1.3)	1.5 (1.2–1.7)	*0.18*

Data are presented as mean±SD or median and IQR.

BSA, body surface area; CI, cardiac index (cardiac output indexed to BSA); LVM ED, LV mass in end diastole; LV EDVi, LV end diastolic volume indexed to BSA; LV ESVi, LV end systolic volume indexed to BSA; LVEF, LV ejection fraction; LVMi, LV mass indexed to BSA; MBF, myocardial blood flow; MPR, myocardial perfusion reserve; SVi, stroke volume indexed to BSA; TAVI, transcatheter aortic valve implantation.

## Discussion

**Figure 1:**
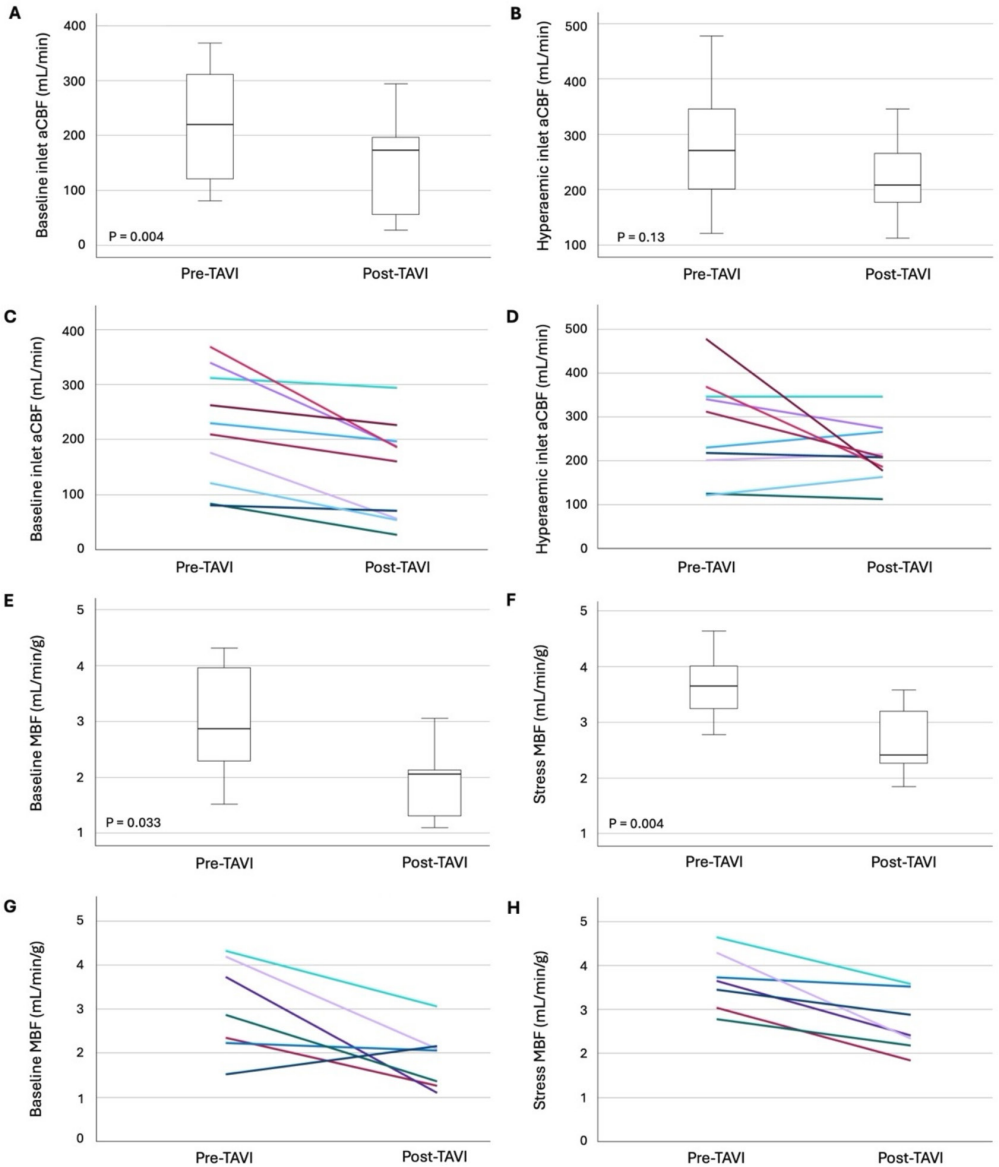
Changes in coronary physiology pre- and 6 months post-TAVI. (A,B) Box and whisker plots comparing mean coronary inlet aCBF values at rest and hyperaemia, respectively. (**C**,**D**) Patient-level individual values at rest and hyperaemia, respectively. (E,F) Box and whisker plots comparing changes in CMR-derived MBF (mL/min/g) pre- and 6 months post-TAVI at rest and hyperaemia, respectively. (G,H) Patient-level individual values for MBF at rest and hyperaemia, respectively. aCBF, absolute coronary blood flow, CMR, cardiac magnetic resonance; MBF, myocardial blood flow; TAVI, transcatheter aortic valve implantation.

**Figure 2:**
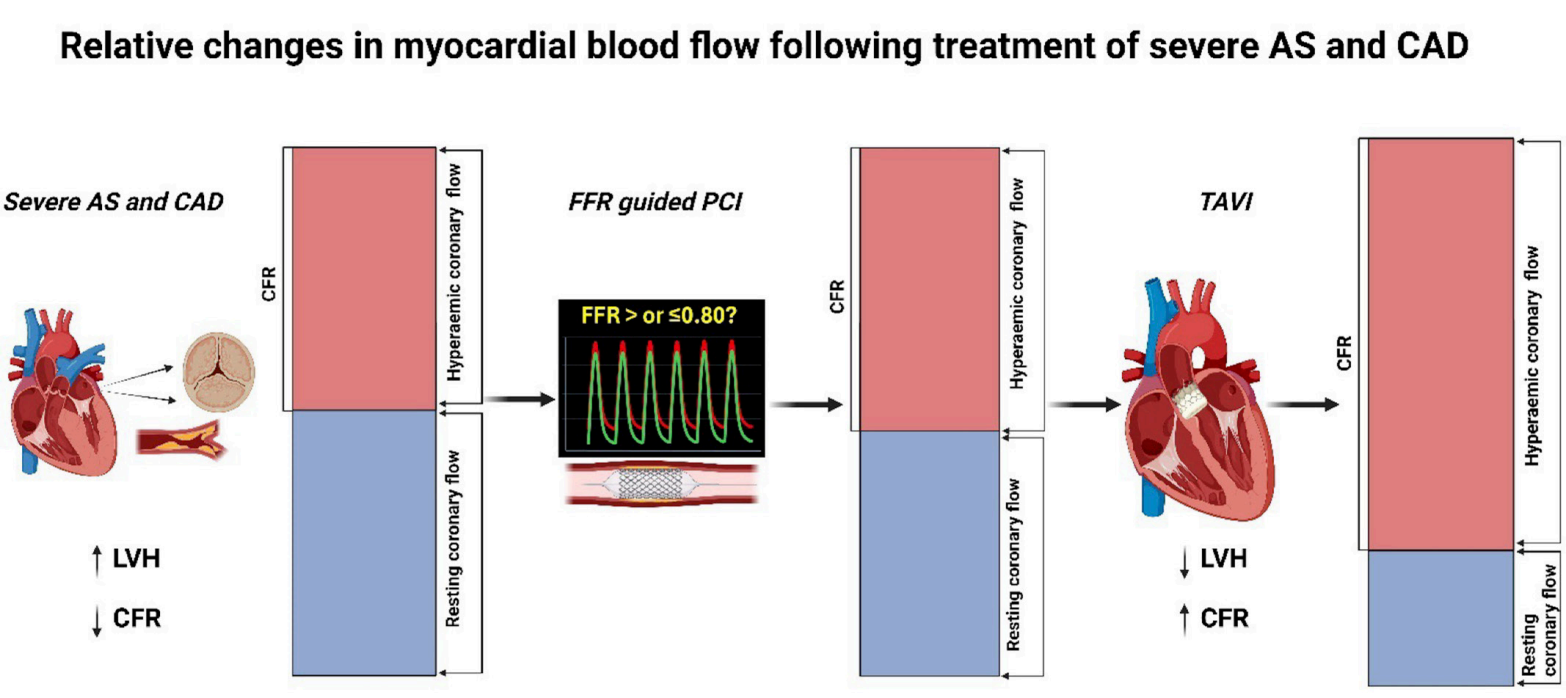
Relative changes in myocardial blood flow following treatment of severe AS and CAD with FFR-guided PCI and subsequent TAVI. AS, aortic stenosis; CAD, coronary artery disease; CFR, coronary flow reserve; FFR, fractional flow reserve; LVH, left ventricular hypertrophy; PCI, percutaneous coronary intervention; TAVI, transcatheter aortic valve implantation.

This study explored the impact of TAVI on MBF and MVD using advanced invasive, computational and imaging techniques. It is among the first to investigate CMR- and CFD-derived indices of MBF and MVD in patients with severe AS and CAD undergoing TAVI. Our main findings were: (1) when measured using CFD (on a vessel specific basis), resting aCBF fell by 33% post-TAVI but hyperaemic flow remained similar (figure 1); (2) myocardial blood flow fell by 37% and 25%, respectively, both at rest and during hyperaemia, when measured using stress perfusion CMR (figure 1); (3) these changes were not due to changes in MVR; (4) there was a significant improvement in CFR, FFR and MRR at follow-up (table 2); (5) there was significant positive LV remodelling post-TAVI.

For the first time, CFD software has been used to model coronary blood flow and MVR simultaneously in patients with coronary disease and severe AS undergoing TAVI. These data were compared with CMR and invasively derived data to provide an array of patient level changes which occur after TAVI. An understanding of the changes that occur in the myocardial vasculature after TAVI in patients with CAD who are considered for PCI is crucial in guiding treatments and streamlining patient pathways.^26^

### Myocardial blood flow

Resting aCBF across all vessels (seven LAD, two RCA, one LCx) decreased by 33%, from 218±90 mL/min pre-TAVI to 146±89 mL/min post-TAVI (p=0.004) (figure 1). However, hyperaemic aCBF remained similar at follow-up (273±104 mL/min vs 215±66 mL/min, p=0.13). These findings align with Sabbah *et al*, who also observed no significant change in invasively measured (using bolus thermodilution) hyperaemic flow in 34 patients post-TAVI or surgical aortic valve replacement (SAVR).^9^ Gallinoro *et al* measured resting and hyperaemic aCBF in 51 patients before and after TAVI and did not find any significant change in resting flows at follow-up but with an increase in hyperaemic values at 6 months.^27^ The net effect is again an increase in demand-related myocardial perfusion, although mechanistically slightly different to the results of our study. Paolisso *et al* and Gutierrez-Barrios *et al* also both used continuous thermodilution to measure aCBF and found that resting aCBF was significantly higher in patients with severe AS compared with controls, while hyperaemic flow remained similar^1^ and mirrored our findings more closely. They also implicated these differences as the cause for impaired vasodilatory capacity and reduced CFR and MRR in patients with severe AS.

When measured using CMR stress perfusion imaging, there were similar significant reductions in MBF at rest and hyperaemia (table 3). This reduction was apparent in both absolute MBF and MBF indexed to LVM. Resting MBF fell from 3.0±0.98 mL/min/g to 1.9±0.7 mL/min/g (p=0.033), representing a 37% reduction at follow-up. Hyperaemic flow fell by 25% from 3.6±0.57 mL/min/g to 2.7±0.7 mL/min/g (p=0.004) (figure 1). Thus, although both indices demonstrate a significant reduction following TAVI, the relative reduction in resting flow is 12% higher than at stress and demonstrates part of the mechanism for improved hyperaemic vasodilatory capacity and CFR. The most obvious explanation for the reduction in MBF is positive LV remodelling demonstrated on CMR, with a 13% reduction in LVMi (table 3). At follow-up, the significant regression of LVH resulted in a myocardium that was much less demanding of blood. However, as we have demonstrated, MBF also fell when indexed to LVM and therefore this does not provide the complete explanation.

The improvements in invasive CFR, MRR and FFR corroborate improvements in relative hyperaemic capacity when myocardial oxygen demand rises, despite a reduction in raw aCBF and MBF values post-TAVI (figure 2). The mechanisms behind this may be related to a reversal of the hostile conditions which typify AS and include systolic flow reversal, relative capillary paucity, high LVEDP, reduced diastolic coronary filling time and high extravascular compressive forces.^28^ In our small cohort of patients, PCI had no effect on CFR but TAVI was seemingly beneficial in producing a myocardium that was less vulnerable to microvascular ischaemia. Our CMR findings are particularly informative as an independent and highly accurate measure of MBF and coronary perfusion, and our results, although small in number and statistically underpowered, are reassuringly mirrored by studies utilising invasive continuous thermodilution, considered to be the gold standard of measuring aCBF.^1 27 29^

Our findings must be viewed within the confines of this small, exploratory study, and no firm conclusions can be drawn on the isolated causative effect of TAVI or PCI on aCBF or CFR. Furthermore, it has to be noted that MBF and CFR are affected by other modifiable factors such as heart rate, blood pressure, LV function and medical therapies. Although there were only minor changes in these parameters in our cohort before and after TAVI (table 1), their role may be under-appreciated by the design and scope of this study.

### Microvascular blood flow

MVR (mm Hg.min/g) and IMR (mm Hg.sec) did not change from baseline to post-PCI or 6 months post-TAVI. These findings align with those of Sabbah *et al*, who noted IMR to remain similar at follow-up despite improvements in CFR and LVMi post-TAVI/SAVR.^9^ Lumley *et al*^30^ and Nishi *et al*^31^ both found invasively measured IMR in patients with severe AS to be similar to controls, indicating that the major cause of ischaemia and impaired physiological reserve in severe AS is likely due to reduced vasodilatory capacity and capillary rarefaction, rather than high MVR. The fact that IMR and MVR remained similar pre- and post-TAVI is somewhat surprising, because MVR is expected to be high due to extravascular compressive forces acting on the microcirculation in severe AS.^26^ The reason for this theoretical discrepancy may be explained by the inability of the microvasculature to proliferate adequately in line with increasing LV mass associated with severe AS.^32^ Rarefaction of the capillary bed in comparison with LV mass may precondition an LV that is more efficient at extracting oxygen from the microcirculation, ameliorating the need for high MVR to extract sufficient oxygen.^9^ Therefore, MVR remains similar and does not change. Instead, increased myocardial oxygen demand is provided by a reduction in the CFR.^9^ In this context, baseline MVR and IMR would be expected to be low rather than high, but the relatively normal IMR measured here and in other studies suggests that a minimal level of resistance is reached in severe AS, which does not fall further, but still allows almost maximal recruitment of the capillary bed. Changes in myocardial blood flow after treatment of severe AS are therefore not necessarily driven by changes in MVR, but through improved efficiency of coronary and microvascular blood flow and reversal of pathophysiological mechanisms of cardiac-coronary coupling.^30^ Recording MVR consistently and reproducibly is notably fraught with difficulty, and, as with MBF, it is also affected by modifiable factors, including medications and LV function. As such, especially given the small study numbers, the results should be interpreted with some caution.

### Clinical implications

The issue of physiological assessment of co-existing CAD in patients undergoing TAVI remains contentious, and many studies have sought to provide insights.^8^ The relationship between visual assessment and haemodynamic significance of coronary stenoses is poor.^33 34^ Furthermore, the haemodynamic conditions of severe AS may lead to underestimation of lesion severity when FFR is used but overestimation when resting indices such as the instantaneous wave free ratio (iFR) or RFR are used.^35^ Our findings are similar to Scarsini *et al* and Sabbah *et al* who noted positive mean RFR/iFR values (≤0.89) when measured in patients with severe AS despite angiographically non-significant CAD and negative mean FFR (>0.80) on the same lesions.^6 7 35^ Resting indices are therefore seemingly vulnerable to the baseline hyperaemic conditions of severe AS. This is relevant because we have demonstrated that resting flows universally (and quite significantly) reduce post-TAVI, which may lead to a wider degree of lesion re-classification post-TAVI. Conversely, vessel specific hyperaemic aCBF (the parameter on which FFR is based) remained similar at follow-up.

### Limitations

This study was exploratory and hypothesis-generating with a small sample size. Although the target of 20 patients was in line with similar studies,^14^ attrition resulted in only seven full data sets which left the results vulnerable to selection bias and weak statistical power. As a single-centre study, it is inherently vulnerable to selection bias and lacks external validation. The results cannot, therefore, be generalised across the entire population of patients with AS, although results in just seven patients seem to show consistent results. Also, a 6-month follow-up, while consistent with similar studies, may be insufficient for capturing long-term myocardial remodelling and haemodynamic improvements. Further to this, not all patients had PCI at the exact same time-point in their pathway towards TAVI, and this may have affected remodelling and MFB results. The use of virtuQ, although previously validated,^15 36^ most recently in a multicentre cohort against the Rayflow continuous infusion catheter (Hexacath, Fr),^37^ is not approved for informing clinical decisions, and over-interpretation of the results should be avoided. Further work may wish to formally assess sensitivity of the virtuQ system.^38^

## Conclusion

In patients with severe AS and CAD, TAVI decreased LVMi, myocardial oxygen demand and aCBF measured under both baseline and hyperaemic conditions (figure 2). Our findings suggest that the reduction in LVMi, aCBF and global MBF—particularly at rest—are the key mechanisms underlying the observed improvement in CFR. These findings are supported by other studies in the field who have used the current gold standard measure of aCBF (continuous thermodilution) and mirror both our invasive and CMR derived findings.^1 29^

## Supplementary material

10.1136/openhrt-2025-003621 online supplemental file 1

## Data Availability

Data are available upon reasonable request.

## References

[1] Paolisso P, Gallinoro E, Vanderheyden M (2022). Absolute coronary flow and microvascular resistance reserve in patients with severe aortic stenosis. Heart.

[2] Johnson NP, Gould KL, De Bruyne B (2021). Autoregulation of Coronary Blood Supply in Response to Demand: JACC Review Topic of the Week. J Am Coll Cardiol.

[3] Gould KL, Carabello BA (2003). Why angina in aortic stenosis with normal coronary arteriograms?. Circulation.

[4] Del Buono MG, Montone RA, Camilli M (2021). Coronary Microvascular Dysfunction Across the Spectrum of Cardiovascular Diseases: JACC State-of-the-Art Review. J Am Coll Cardiol.

[5] Ahn J-H, Kim SM, Park S-J (2016). Coronary Microvascular Dysfunction as a Mechanism of Angina in Severe AS: Prospective Adenosine-Stress CMR Study. J Am Coll Cardiol.

[6] Scarsini R, Pesarini G, Zivelonghi C (2018). Physiologic evaluation of coronary lesions using instantaneous wave-free ratio (iFR) in patients with severe aortic stenosis undergoing transcatheter aortic valve implantation. EuroIntervention.

[7] Sabbah M, Joshi FR, Minkkinen M (2022). Long-Term Changes in Invasive Physiological Pressure Indices of Stenosis Severity Following Transcatheter Aortic Valve Implantation. Circ Cardiovasc Interv.

[8] Minten L, Bennett J, Otsuki H (2024). Differential Effect of Aortic Valve Replacement for Severe Aortic Stenosis on Hyperemic and Resting Epicardial Coronary Pressure Indices. J Am Heart Assoc.

[9] Sabbah M, Olsen NT, Holmvang L (2023). Long-term changes in coronary physiology after aortic valve replacement. EuroIntervention.

[10] Mack MJ, Leon MB, Smith CR (2015). 5-year outcomes of transcatheter aortic valve replacement or surgical aortic valve replacement for high surgical risk patients with aortic stenosis (PARTNER 1): a randomised controlled trial. The Lancet.

[11] Leon MB, Smith CR, Mack MJ (2016). Transcatheter or Surgical Aortic-Valve Replacement in Intermediate-Risk Patients. N Engl J Med.

[12] Patterson T, Clayton T, Dodd M (2021). ACTIVATION (PercutAneous Coronary inTervention prIor to transcatheter aortic VAlve implantaTION): A Randomized Clinical Trial. JACC Cardiovasc Interv.

[13] Lønborg J, Jabbari R, Sabbah M (2024). PCI in Patients Undergoing Transcatheter Aortic-Valve Implantation. N Engl J Med.

[14] Yones E, Gunn J, Iqbal J (2025). Functional assessment of coronary artery disease in patients with severe aortic stenosis: a review. Heart.

[15] Morris PD, Gosling R, Zwierzak I (2021). A novel method for measuring absolute coronary blood flow and microvascular resistance in patients with ischaemic heart disease. Cardiovasc Res.

[16] Beyersdorf F, Vahanian A, Milojevic M (2022). Corrigendum to: 2021 ESC/EACTS Guidelines for the management of valvular heart disease. Eur Heart J.

[17] Neumann F-J, Sousa-Uva M, Ahlsson A (2019). 2018 ESC/EACTS Guidelines on myocardial revascularization. EuroIntervention.

[18] Pijls NH, De Bruyne B, Peels K (1996). Measurement of fractional flow reserve to assess the functional severity of coronary-artery stenoses. N Engl J Med.

[19] De Bruyne B, Pijls NHJ, Gallinoro E (2021). Microvascular Resistance Reserve for Assessment of Coronary Microvascular Function: JACC Technology Corner. J Am Coll Cardiol.

[20] Aubiniere-Robb L, Gosling R, Taylor DJ (2022). The Complementary Value of Absolute Coronary Flow in the Assessment of Patients with Ischaemic Heart Disease (the COMPAC-Flow Study). *Nat Cardiovasc Res*.

[21] Taylor DJ, Feher J, Czechowicz K (2023). Validation of a novel numerical model to predict regionalized blood flow in the coronary arteries. European Heart Journal - Digital Health.

[22] Huo Y, Kassab GS (2012). Intraspecific scaling laws of vascular trees. J R Soc Interface.

[23] Taylor DJ, Saxton H, Halliday I (2024). Systematic review and meta-analysis of Murray’s law in the coronary arterial circulation. Am J Physiol Heart Circ Physiol.

[24] Zhou W, Sin J, Yan AT (2023). Qualitative and Quantitative Stress Perfusion Cardiac Magnetic Resonance in Clinical Practice: A Comprehensive Review. Diagnostics (Basel).

[25] Brown LAE, Onciul SC, Broadbent DA (2018). Fully automated, inline quantification of myocardial blood flow with cardiovascular magnetic resonance: repeatability of measurements in healthy subjects. J Cardiovasc Magn Reson.

[26] Zelis JM, Tonino PAL, Pijls NHJ (2020). Coronary Microcirculation in Aortic Stenosis: Pathophysiology, Invasive Assessment, and Future Directions. J Interv Cardiol.

[27] Gallinoro E, Paolisso P, Bertolone DT (2024). Absolute coronary flow and microvascular resistance before and after transcatheter aortic valve implantation. EuroIntervention.

[28] McConkey HZR, Marber M, Chiribiri A (2019). Coronary Microcirculation in Aortic Stenosis. Circ: Cardiovascular Interventions.

[29] Gutiérrez-Barrios A, Cañadas-Pruaño D, Alfaro LM (2024). Coronary Flow Reserve and Myocardial Resistance Reserve Changes After Transcatheter Aortic Valve Implantation in Aortic Stenosis. Am J Cardiol.

[30] Lumley M, Williams R, Asrress KN (2016). Coronary Physiology During Exercise and Vasodilation in the Healthy Heart and in Severe Aortic Stenosis. J Am Coll Cardiol.

[31] Nishi T, Kitahara H, Saito Y (2018). Invasive assessment of microvascular function in patients with valvular heart disease. Coron Artery Dis.

[32] Mahmod M, Chan K, Raman B (2019). Histological Evidence for Impaired Myocardial Perfusion Reserve in Severe Aortic Stenosis. JACC: Cardiovascular Imaging.

[33] Williams GJ, Taylor DJ, Al Baraikan A (2025). Virtual physiological analysis of non-culprit disease in patients with STEMI and multivessel disease: a substudy of the COMPLETE trial. Eur Heart J Open.

[34] Taylor DJ, Saxton H, Xu X (2025). Invasive validation of novel 1D models for computation of coronary fractional flow reserve. Cardiovasc Res.

[35] Scarsini R, Lunardi M, Venturi G (2020). Long-term variations of FFR and iFR after transcatheter aortic valve implantation. Int J Cardiol.

[36] Taylor DJ, Feher J, Halliday I (2022). Refining Our Understanding of the Flow Through Coronary Artery Branches; Revisiting Murray’s Law in Human Epicardial Coronary Arteries. Front Physiol.

[37] Taylor DJ, Newman T, Tlałka K Validation of a physics-based computational model of epicardial and microvascular coronary physiology against continuous infusion thermodilution. Cardiovascular Medicine.

[38] Saxton H, Taylor DJ, Xu X (2025). Derivation and sensitivity analysis of a novel one-dimensional model of coronary blood flow accounting for vessel taper and boundary slip. Am J Physiol Heart Circ Physiol.

