# Supplementary material for: Changes in myocardial blood flow and microvascular resistance in patients with coronary artery disease undergoing transcatheter aortic valve implantation
Source: Open Heart. 2025 Dec 30;12(2):e003621. doi: 10.1136/openhrt-2025-003621 (PMC12766762; doi:10.1136/openhrt-2025-003621)
Supplement: online supplemental file 1 [file openhrt-12-2-s001.docx]

# Supplementary images

# **
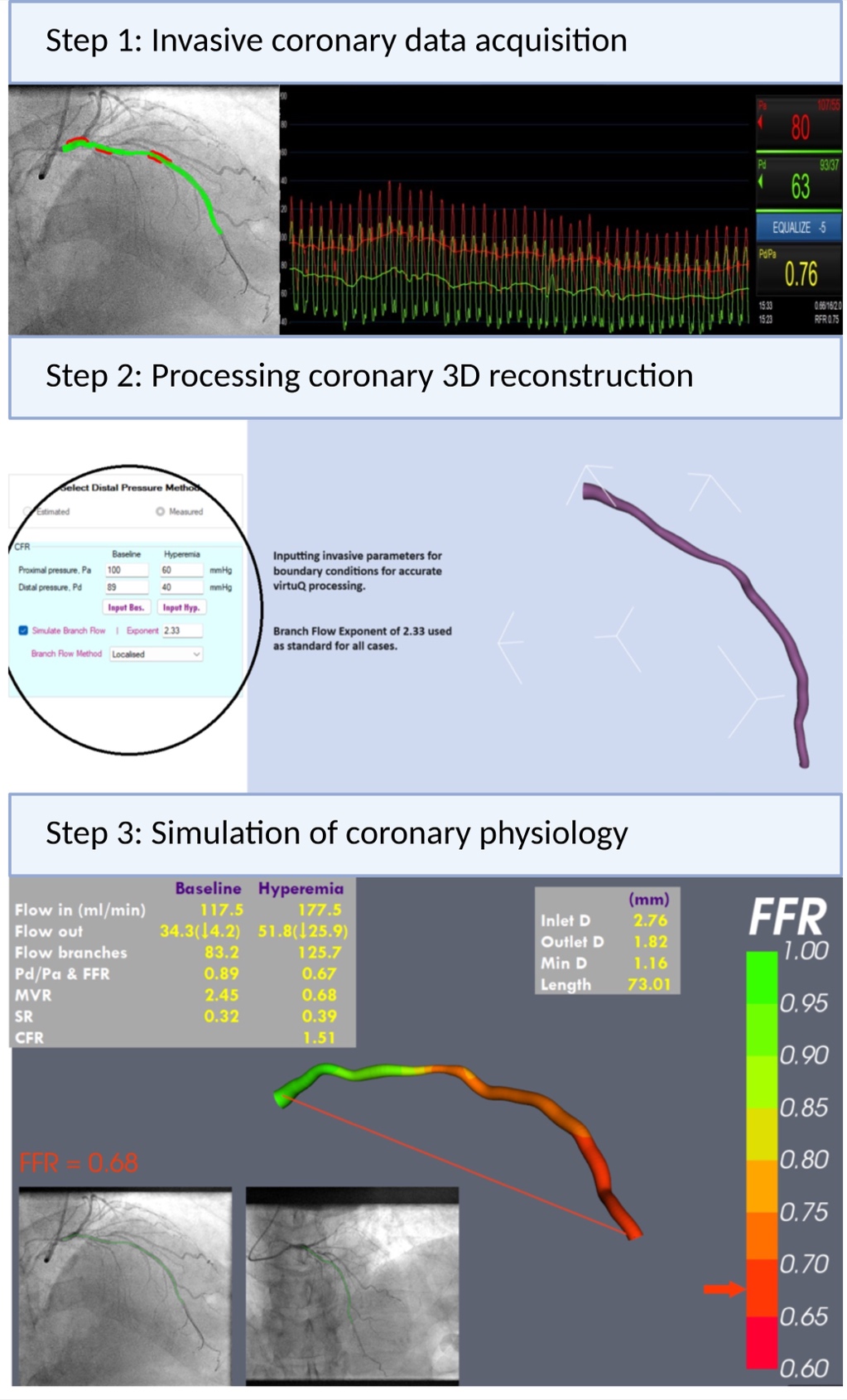
**

# Supplementary figure 1. Schematic representation of virtuQ workflow.


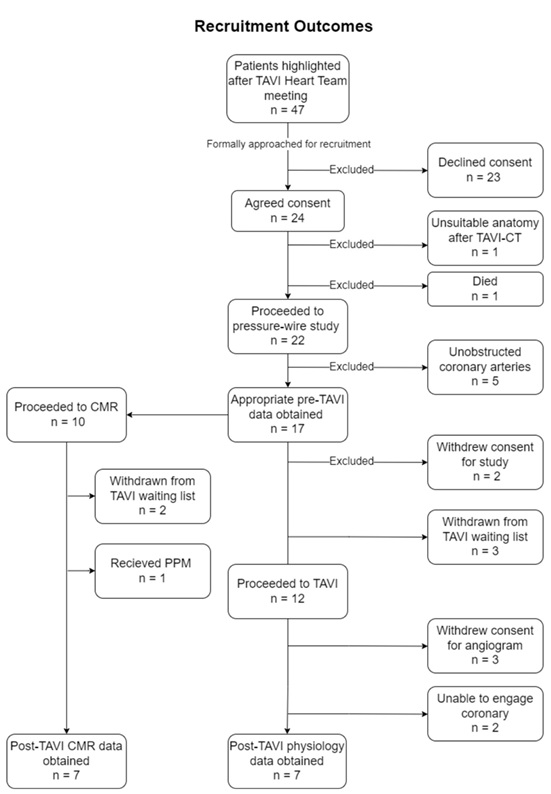


**Supplementary figure 2. Consort diagram.**
